# Effects of proton therapy on regional [^18^F]FDG uptake in non-tumor brain regions of patients treated for head and neck cancer

**DOI:** 10.1016/j.ctro.2023.100652

**Published:** 2023-06-19

**Authors:** Wejdan M. Arif, Philip H. Elsinga, Roel J.H.M. Steenbakkers, Walter Noordzij, Lara Barazzuol, Kelvin N.G. Wei Siang, Charlotte L. Brouwer, Bruno Lima Giacobbo, Rudi A.J.O. Dierckx, Ronald J.H. Borra, Gert Luurtsema

**Affiliations:** a University of Groningen, University Medical Center Groningen, Department of Nuclear Medicine and Molecular Imaging, Hanzeplein 1, 9713 GZ Groningen, the Netherlands; b King Saud University, College of Applied Medical Science, Department of Radiological Sciences, Riyadh, Saudi Arabia; c University of Groningen, University Medical Center Groningen, Department of Radiation Oncology, Hanzeplein 1, 9713 GZ Groningen, the Netherlands; d University of Groningen, University Medical Center Groningen, Department of Biomedical Sciences of Cells and Systems, Hanzeplein 1, 9713 GZ Groningen, the Netherlands

**Keywords:** Glucose metabolism, PET, Head and neck cancer, NPC, IMPT, Radiation dose

## Abstract

**Background and purpose:**

Previous pre-clinical research using [^18^F]FDG-PET has shown that whole-brain photon-based radiotherapy can affect brain glucose metabolism. This study, aimed to investigate how these findings translate into regional changes in brain [^18^F]FDG uptake in patients with head and neck cancer treated with intensity-modulated proton therapy (IMPT).

**Materials and methods:**

Twenty-three head and neck cancer patients treated with IMPT and available [^18^F]FDG scans before and at 3 months follow-up were retrospectively evaluated. Regional assessment of the [^18^F]FDG standardized uptake value (SUV) parameters and radiation dose in the left (L) and right (R) hippocampi, L and R occipital lobes, cerebellum, temporal lobe, L and R parietal lobes and frontal lobe were evaluated to understand the relationship between regional changes in SUV metrics and radiation dose.

**Results:**

Three months after IMPT, [^18^F]FDG brain uptake calculated using SUVmean and SUVmax, was significantly higher than that before IMPT. The absolute SUVmean after IMPT was significantly higher than before IMPT in seven regions of the brain (p ≤ 0.01), except for the R (p = 0.11) and L (p = 0.15) hippocampi. Absolute and relative changes were variably correlated with the regional maximum and mean doses received in most of the brain regions.

**Conclusion:**

Our findings suggest that 3 months after completion of IMPT for head and neck cancer, significant increases in the uptake of [^18^F]FDG (reflected by SUVmean and SUVmax) can be detected in several individual key brain regions, and when evaluated jointly, it shows a negative correlation with the mean dose. Future studies are needed to assess whether and how these results could be used for the early identification of patients at risk for adverse cognitive effects of radiation doses in non-tumor tissues.

## Highlights

• [^18^F]FDG-PET can be used to monitor changes in glucose metabolism by most brain regions before and after IMPT in NPC.
• Three months after IMPT for NPC, a significant increases in the uptake of [^18^F]FDG by most brain regions was observed.
• A negative correlation was shown between FDG uptake and radiation dose in several brain regions.
• Changes in the SUV appear to be dependent on the radiation dose in some brain regions.

## Introduction

Head and neck cancer is one of the most common forms of cancer worldwide [1]. This type of cancer can be treated by surgery, radiotherapy (RT), systemic treatment, or a combination of these treatments, depending on the type of tumor, its location and stage, the patient’s condition, and the availability of treatment modalities in the relative hospital. In general, treating head and neck cancer using radiation therapy is challenging. This is due to the proximity of the tumor to critical organs, heterogeneity of the surrounding tissue, and the possibility of anatomical changes during therapy [2], [3]. Patients with nasopharyngeal carcinoma (NPC) often develop cognitive decline due to radiation doses (>10 Gy) received by the bilateral temporal lobes [4], [5], [6]. This brain region usually receives radiation, because it is in close proximity to the clinical target volume in conventional nasopharyngeal radiotherapy treatment plans. Moreover, scattered radiation can affect other brain regions [6], [7]. In this regard, the relationship between irradiation and brain injury that causes cognitive decline is still poorly understood. Preclinical and clinical studies have correlated cognitive dysfunction with cerebral blood flow disturbance [8], [9], [10]. A strong correlation has been observed between the severity of cognitive deficits and the extent of hypoperfusion [8]. Similarly, alterations in glucose metabolism are also one of the side effects that can appear in the long term [11], [12]. Changes in [^18^F] fluorodeoxyglucose ([^18^F]FDG) uptake were observed in adult and pediatric patients with brain cancer who received long-term external beam cranial radiation, as shown by positron emission tomography (PET) scans.

The cerebral metabolic rate of glucose in non-tumor brain regions that received radiation doses (>10 Gy) was low compared to healthy subjects and cancer patients who underwent surgery alone [11], [12]. The reason for this finding remains unclear with some researchers speculating that it is linked to changes in cerebral blood flow and damage to the white matter parenchyma [13]. A preclinical study published by Parente et al. [14] in 2020 evaluated the early- delayed effect of cranial irradiation on days 3 and 31 using [^18^F]FDG PET and found that cranial irradiation at 10 Gy led to increased [^18^F]FDG uptake; in contrast, 25 Gy resulted in a decreased in [^18^F]FDG uptake. This suggests that changes in brain glucose metabolism vary depending on the radiation dose received [15], [16]. A study by Hahn et al. in a group of six patients with CNS tumors evaluated the effects of photon radiotherapy using both [^18^F]FDG and [^15^O]H_2_O PET imaging at 3 weeks and 6 months in anatomical areas corresponding to 5 Gy dose bins. They found that in regions receiving more than 40 Gy a decreased glucose metabolism which was correlated with decreased performance in neuropsychological tests [16]. The aforementioned study emphasized the potential importance of [^18^F]FDG PET imaging in detecting patients prone to cognitive decline.

Unlike previous studies on photon therapy, to the best of our knowledge, no similar data exist regarding the effects of proton therapy applied to patients with head and neck cancers (e.g., NFC), where it is expected that there should be no significant direct effects of the tumor itself on brain tissue metabolism. Information on changes in [^18^F]FDG PET uptake in anatomical brain areas, including areas relevant to cognition, could provide valuable information to a better understand the impact of this therapy modality on cognition solely due to proton therapy. Therefore, the current study aimed to take the first step toward this goal by investigating the effect of intensity-modulated proton therapy (IMPT) on changes in regional glucose metabolism in the brain, evaluated using [^18^F]FDG-PET.

## Materials and methods

### Patient selection

Twenty three patients clinically diagnosed with nasopharyngeal cancer and treated with IMPT were retrospectively included in this study between January 2018 and January 2022. Patient characteristics, including sex, age, tumor location and the histology are displayed in Table 1.

**Table 1:** Patient and tumor characteristics.

**Patient characteristics**		
**characteristics**		**Total (n = 23)**
**Sex**		**n**
	Male	16
	Female	7

**Age**		**Years**
	Mean (standard deviation)	53.5 (32)
	Median (range)	46.5 (24–74)
	Interquartile range	65.5–48 = 17.5

**Histology**		**n**
	Carcinoma	20
	Squamous cell carcinoma	3

**Tumor location**		**n**
	Nasopharynx	22
	Maxillary sinus right	1

### PET/CT imaging

All [^18^F]FDG PET scans were performed using a Siemens Biograph mCT 64-slice PET/CT scanner (Siemens Healthineers, Knoxville, TN, USA), and a 128-slice Siemens Biograph Vision scanner (Siemens Healthineers) with 60 min of uptake time. Participants were instructed to fast overnight for at least six h. Low-dose CT imaging was performed to visualize anatomical structures and was used as an attenuation correction map. PET acquisitions were obtained at 1.5 min per bed for <60 kg, 2 min per bed for 60–90 kg, and 3 min per bed position for >90 kg.

### Imaging and analysis

**Fig. 1:**
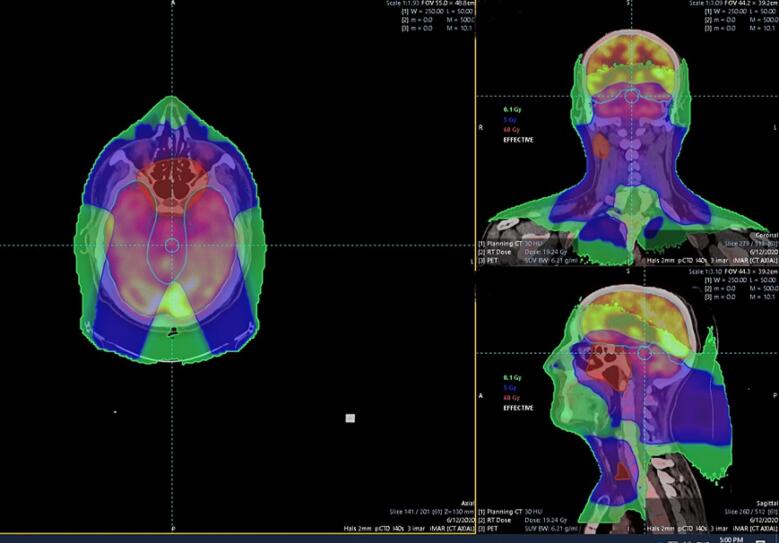
Proton therapy plan and PET imaging in nasopharyngeal carcinoma patient.

All patients underwent IMPT and received two [^18^F]FDG-PET scans, one before receiving IMPT and the second three months after completion the IMPT. All of them provided informed consent to use their data for research purposes. A contrast- enhanced computed tomography (CT) scan of the head and neck with a 2 mm slice thickness was performed for all the patients, which was required for radiotherapy planning. For the PET scan, the European Association of Nuclear Medicine Research Ltd. (EARL) reconstruction was used to determine the maximum standardized uptake value (SUVmax) within predefined regions of interest in the brain related to cognition, taking into consideration the glucose level before tracer injection. The regions of interest were the left (L) and right (R) hippocampi, L and R occipital lobes, cerebellum, temporal lobe, L and R parietal lobes and frontal lobe. IMPT doses and IMPT structures were retrieved from the hospital Picture Archiving and Communication System (PACS) into Mirada DBx 1.2 (Mirada Medical Ltd, Oxford, UK). All data underwent image registration and automated segmentation using process by multi-atlas segmentation (Mirada RTx Advanced 1.8 & Workflow Box 2.0, Mirada Medical Ltd, Oxford, UK), in which the aforementioned brain regions, PET and CT images, and radiation doses were registered and fused (Fig. 1). The SUVmean and SUVmax as well as the mean and maximum cumulative radiation doses for the different brain regions were subsequently recorded. We then performed a regional assessment, where the SUVmean before and after IMPT was evaluated, followed by correlation of SUVmax and SUVmean with the mean dose obtained by each region for each individual patient.

### Statistical analysis

[^18^F]FDG uptake was normalized to the weight and injected dose to obtain the standardized uptake value (SUV). For each brain region, SUV measurements obtained before and after proton therapy were compared using the generalized estimating equation (GEE) to evaluate the main effect of radiotherapy on [^18^F]FDG uptake. To assess whether SUV values are correlated to the dose administered to the patient during proton therapy sessions, a correlation analysis was performed using Pearson correlation by subtracting the mean and maximum SUV values from before and after proton therapy. For all analyses, a p-value <0.05 was considered statistically significant. All analyses were performed using IBM SPSS package version 23.0 (IBM, Armonk, NY, USA).

## Results

**Fig. 2:**
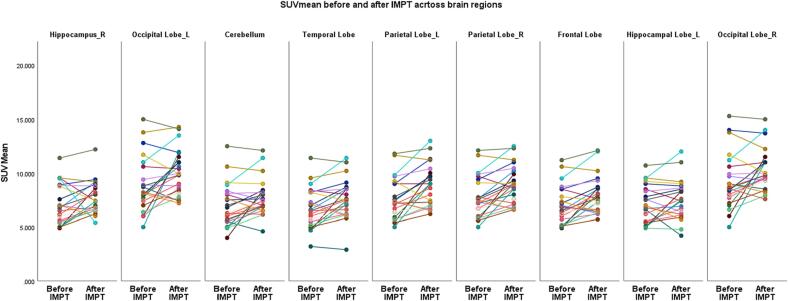
Measurements of SUVmean before and at 3-months follow up after IMPT within the same patient across all assessed brain regions.

An overall significant increase in absolute SUVmean was observed after proton therapy in seven regions of the brain except for the R and L hippocampi. An overview of the mean value differences before and at 3 months of follow-up after proton therapy for the assessed brain regions. The respective statistical significances for SUVmean and SUVmax are shown in Table 2a, Table 2b. SUVmean measurements before and at 3 months of follow-up after IMPT in the same patient in all regions of the brain are shown in Fig. 2. Furthermore, when evaluating the assessed brain regions and considering them together, the SUVmean values were significantly and negatively correlated with the mean dose for all the assessed regions (p = 0.0001).

**Table 2a:** SUVmean before and after IMPT at 3-month follow-up and the statistical significance of the changes in the assessed brain regions.

Region = Frontal Lobe							
Timepoint	Mean	Std. Error	95% Wald Confidence Interval				
			Lower	Upper			
Before	7.07	0.36	6.36	7.77			
After	7.99	0.35	7.30	8.68			
Pairwise Comparisons							

(I) Timepoint	(J) Timepoint	Mean Difference (I-J)	Std. Error	df	Bonferroni Sig.	95% Wald Confidence Interval for Difference	
						Lower	Upper
Before	After	−0.92	0.26	1	0	−1.43	−0.41
After	Before	0.92	0.26	1	0	0.41	1.43

Overall Test Resultsa							
Wald Chi-Square	df	Sig.					
12.57	1	0					

Region = Temporal Lobe							

Timepoint	Mean	Std. Error	95% Wald Confidence Interval				
			Lower	Upper			
Before	6.74	0.38	5.99	7.50			
After	7.58	0.38	6.84	8.32			
Pairwise Comparisons							

(I) Timepoint	(J) Timepoint	Mean Difference (I-J)	Std. Error	df	Bonferroni Sig.	95% Wald Confidence Interval for Difference	
						Lower	Upper
Before	After	−0.83	0.25	1	0.00	−1.33	−0.33
After	Before	0.83	0.25	1	0.00	0.33	1.33

Overall Test Resultsa							
Wald Chi-Square	df	Sig.					
10.65	1.00	0.00					

Region = Cerebellum							

Timepoint	Mean	Std. Error	95% Wald Confidence Interval				
			Lower	Upper			
Before	6.97	0.41	6.17	7.77			
After	7.75	0.34	7.08	8.43			
Pairwise Comparisons							

(I) Timepoint	(J) Timepoint	Mean Difference (I-J)	Std. Error	df	Bonferroni Sig.	95% Wald Confidence Interval for Difference	
						Lower	Upper
Before	After	−0.78	0.28	1.00	0.01	−1.33	−0.23
After	Before	0.78	0.28	1.00	0.01	0.23	1.33

Overall Test Resultsa							
Wald Chi-Square	df	Sig.					
7.77	1.00	0.01					

Region = R Parietal Lobe							

Timepoint	Mean	Std. Error	95% Wald Confidence Interval				
			Lower	Upper			
Before	7.78	0.39	7.00	8.55			
After	8.91	0.36	8.21	9.62			
Pairwise Comparisons							

(I) Timepoint	(J) Timepoint	Mean Difference (I-J)	Std. Error	df	Bonferroni Sig.	95% Wald Confidence Interval for Difference	
						Lower	Upper
Before	After	−1.13	0.29	1.00	0.00	−1.71	−0.56
After	Before	1.13	0.29	1.00	0.00	0.56	1.71

Overall Test Resultsa							
Wald Chi-Square	df	Sig.					
15.02	1	0					

Region = L Parietal Lobe							

Timepoint	Mean	Std. Error	95% Wald Confidence Interval				
			Lower	Upper			
Before	7.62	0.39	6.86	8.38			
After	8.81	0.40	8.04	9.59			
Pairwise Comparisons							

(I) Timepoint	(J) Timepoint	Mean Difference (I-J)	Std. Error	df	Bonferroni Sig.	95% Wald Confidence Interval for Difference	
						Lower	Upper
Before	After	−1.19	0.32	1.00	0.00	−1.83	−0.56
After	Before	1.19	0.32	1.00	0.00	0.56	1.83

Overall Test Resultsa							
Wald Chi-Square	df	Sig.					
13.61	1	0					

Region = R Hippocampus							

Timepoint	Mean	Std. Error	95% Wald Confidence Interval				
			Lower	Upper			
Before	7.12	0.39	6.36	7.89			
After	7.68	0.32	7.05	8.31			
Pairwise Comparisons							

(I) Timepoint	(J) Timepoint	Mean Difference (I-J)	Std. Error	df	Bonferroni Sig.	95% Wald Confidence Interval for Difference	
						Lower	Upper
Before	After	−0.56	0.35	1.00	0.11	−1.26	0.13
After	Before	0.56	0.35	1.00	0.11	−0.13	1.26

Overall Test Resultsa							
Wald Chi-Square	df	Sig.					
2.51	1.00	0.11					

Region = L Hippocampus							

Timepoint	Mean	Std. Error	95% Wald Confidence Interval				
			Lower	Upper			
Before	7.11	0.35	6.43	7.80			
After	7.52	0.37	6.78	8.25			
Pairwise Comparisons							

(I) Timepoint	(J) Timepoint	Mean Difference (I-J)	Std. Error	df	Bonferroni Sig.	95% Wald Confidence Interval for Difference	
						Lower	Upper
Before	After	−0.40	0.28	1.00	0.15	−0.95	0.14
After	Before	0.40	0.28	1.00	0.15	−0.14	0.95

Overall Test Resultsa							
Wald Chi-Square	df	Sig.					
2.11	1	0.15					

Region = R Occipital Lobe							

Timepoint	Mean	Std. Error	95% Wald Confidence Interval				
			Lower	Upper			
Before	9.22	0.52	8.19	10.25			
After	10.34	0.40	9.57	11.12			
Pairwise Comparisons							

(I) Timepoint	(J) Timepoint	Mean Difference (I-J)	Std. Error	df	Bonferroni Sig.	95% Wald Confidence Interval for Difference	
						Lower	Upper
Before	After	−1.12	0.41	1.00	0.01	−1.93	−0.31
After	Before	1.12	0.41	1.00	0.01	0.31	1.93

Overall Test Resultsa							
Wald Chi-Square	df	Sig.					
7.32	1.00	0.01					

Region = L Occipital Lobe							

Timepoint	Mean	Std. Error	95% Wald Confidence Interval				
			Lower	Upper			
Before	8.87	0.52	7.85	9.88			
After	10.11	0.43	9.27	10.94			
Pairwise Comparisons							

(I) Timepoint	(J) Timepoint	Mean Difference (I-J)	Std. Error	df	Bonferroni Sig.	95% Wald Confidence Interval for Difference	
						Lower	Upper
Before	After	−1.24	0.44	1.00	0.01	−2.10	−0.38
After	Before	1.24	0.44	1.00	0.01	0.38	2.10

Overall Test Resultsa							
Wald Chi-Square	df	Sig.					
7.98	1.00	0.01

**Table 2b:** SUVmax before and after IMPT at 3-month follow-up and the statistical significance of the changes in the assessed brain regions.

Region = Frontal Lobe							
Timepoint	Mean	Std. Error	95% Wald Confidence Interval				
			Lower	Upper			
Before	13.04	0.63	11.80	14.27			
After	14.43	0.62	13.21	15.65			
a Region = 7							
Pairwise Comparisons							
(I) Timepoint	(J) Timepoint	Mean Difference (I-J)	Std. Error	df	Bonferroni Sig.	95% Wald Confidence Interval for Difference	
						Lower	Upper
Before	After	−1.39	0.49	1.00	0.00	−2.35	−0.44
After	Before	1.39	0.49	1.00	0.00	0.44	2.35

Overall Test Resultsa							
Wald Chi-Square	df	Sig.					
8.25	1.00	0.00					

Region = Temporal Lobe							
Timepoint	Mean	Std. Error	95% Wald Confidence Interval				
			Lower	Upper			
Before	12.29	0.82	10.68	13.91			
After	14.39	0.76	12.90	15.88			
a Region = 4							
Pairwise Comparisons							
(I) Timepoint	(J) Timepoint	Mean Difference (I-J)	Std. Error	df	Bonferroni Sig.	95% Wald Confidence Interval for Difference	
						Lower	Upper
Before	After	−2.09	0.99	1.00	0.03	−4.04	−0.16
After	Before	2.09	0.99	1.00	0.03	0.16	4.04

Overall Test Resultsa							
Wald Chi-Square	df	Sig.					
4.48	1	0.03					

Region = Cerebellum							
Timepoint	Mean	Std. Error	95% Wald Confidence Interval				
			Lower	Upper			
Before	11.04	0.54	9.98	12.10			
After	12.57	0.48	11.64	13.50			
a Region = 3							
Pairwise Comparisons							
(I) Timepoint	(J) Timepoint	Mean Difference (I-J)	Std. Error	df	Bonferroni Sig.	95% Wald Confidence Interval for Difference	
						Lower	Upper
Before	After	−1.53	0.43	1.00	0.00	−2.37	−0.68
After	Before	1.53	0.43	1.00	0.00	0.68	2.37

Overall Test Resultsa							
Wald Chi-Square	df	Sig.					
12.55	1.00	<0.001					

Region = R Parietal Lobe							
Timepoint	Mean	Std. Error	95% Wald Confidence Interval				
			Lower	Upper			
Before	13.58	0.63	12.34	14.82			
After	15.82	0.72	14.42	17.22			
a Region = 6							
Pairwise Comparisons							
(I) Timepoint	(J) Timepoint	Mean Difference (I-J)	Std. Error	df	Bonferroni Sig.	95% Wald Confidence Interval for Difference	
						Lower	Upper
Before	After	−2.23	0.67	1.00	0.00	−3.54	−0.93
After	Before	2.23	0.67	1.00	0.00	0.93	3.54

Overall Test Resultsa							
Wald Chi-Square	df	Sig.					
11.22	1.00	<0.001					

Region = L Parietal Lobe							
Timepoint	Mean	Std. Error	95% Wald Confidence Interval				
			Lower	Upper			
Before	13.44	0.59	12.28	14.59			
After	15.71	0.69	14.36	17.07			
a Region = 5							
Pairwise Comparisons							
(I) Timepoint	(J) Timepoint	Mean Difference (I-J)	Std. Error	df	Bonferroni Sig.	95% Wald Confidence Interval for Difference	
						Lower	Upper
Before	After	−2.28	0.64	1.00	0.00	−3.53	−1.03
After	Before	2.28	0.64	1.00	0.00	1.03	3.53

Overall Test Resultsa							
Wald Chi-Square	df	Sig.					
12.8	1	<0.001					

Region = R Hippocampus							
Timepoint	Mean	Std. Error	95% Wald Confidence Interval				
			Lower	Upper			
Before	8.57	0.45	7.68	9.45			
After	9.71	0.47	8.80	10.62			
a Region = 1							
Pairwise Comparisons							
(I) Timepoint	(J) Timepoint	Mean Difference (I-J)	Std. Error	df	Bonferroni Sig.	95% Wald Confidence Interval for Difference	
						Lower	Upper
Before	After	−1.14	0.41	1.00	0.01	−1.96	−0.33
After	Before	1.14	0.41	1.00	0.01	0.33	1.96

Overall Test Resultsa							
Wald Chi-Square	df	Sig.					
7.65	1.00	0.01					

Region = L Hippocampus							
Timepoint	Mean	Std. Error	95% Wald Confidence Interval				
			Lower	Upper			
Before	8.56	0.43	7.71	9.41			
After	9.27	0.48	8.33	10.21			
a Region = 8							
Pairwise Comparisons							
(I) Timepoint	(J) Timepoint	Mean Difference (I-J)	Std. Error	df	Bonferroni Sig.	95% Wald Confidence Interval for Difference	
						Lower	Upper
Before	After	−0.71	0.37	1.00	0.06	−1.44	0.02
After	Before	0.71	0.37	1.00	0.06	−0.02	1.44

Overall Test Resultsa							
Wald Chi-Square	df	Sig.					
3.59	1.00	0.06					

Region = R Occipital Lobe							
Timepoint	Mean	Std. Error	95% Wald Confidence Interval				
			Lower	Upper			
Before	14.61	0.79	13.05	16.16			
After	17.10	0.66	15.81	18.40			
a Region = 9							
Pairwise Comparisons							
(I) Timepoint	(J) Timepoint	Mean Difference (I-J)	Std. Error	df	Bonferroni Sig.	95% Wald Confidence Interval for Difference	
						Lower	Upper
Before	After	−2.49	0.72	1.00	0.00	−3.91	−1.08
After	Before	2.49	0.72	1.00	0.00	1.08	3.91

Overall Test Resultsa							
Wald Chi-Square	df	Sig.					
12.00	1	<0.001					

Region = L Occipital Lobe							
Timepoint	Mean	Std. Error	95% Wald Confidence Interval				
			Lower	Upper			
Before	14.26	0.81	12.68	15.84			
After	17.00	0.70	15.63	18.38			
a Region = 2							
Pairwise Comparisons							
(I) Timepoint	(J) Timepoint	Mean Difference (I-J)	Std. Error	df	Bonferroni Sig.	95% Wald Confidence Interval for Difference	
						Lower	Upper
Before	After	−2.74	0.72	1.00	0.00	−4.14	−1.34
After	Before	2.74	0.72	1.00	0.00	1.34	4.14

Overall Test Resultsa							
Wald Chi-Square	df	Sig.					
14.64	1	<0.001

The relationship between SUVmean post-IMPT and mean dose was also independently evaluated for each region, showing a significant negative correlation between SUVmean post-IMPT and mean dose *only* in the temporal lobe (p = 0.03, Table 3a). However, no correlation was found between SUVmax post-IMPT and maximum dose in any of the assessed regions.

**Table 3a:** Correlation between SUV mean- absolute and relative difference with mean dose per assessed region.

**Region**	**SUV mean post IMPT vs. mean dose**	**SUV mean absolute difference (post-pre IMPT) vs. mean dose**	**SUV mean relative difference (post-pre IMPT) vs. mean dose**
**Frontal lobe**	0.34	0.11	*0.05
**Temporal lobe**	*0.03	0.19	0.16
**Cerebellum**	0.14	“0.10	“0.08
**R parietal lobe**	0.82	0.47	0.49
**L parietal lobe**	0.94	*0.01	*0.001
**R hippocampus**	0.49	“0.07	*0.03
**L hippocampus**	0.50	0.15	0.10
**R occipital lobe**	0.69	*0.005	*0.01
**L occipital lobe**	1.00	*0.01	*0.01

P-values for correlation between SUV mean- absolute and relative difference with mean dose per assessed region, (*) considered significant and (“) is close to statistically significant.

When evaluating the absolute and relative difference of SUVmax and SUVmean and correlating these values with max and mean dose across the nine assessed regions independently, we observed a significant correlation between the absolute SUVmean difference and the mean dose in three assessed regions: the L occipital lobe (r (23) = 0.53, p = 0.01), L parietal lobe (r (23) = 0.54, p = 0.01), and R occipital lobe (r (23) = 0.56, p = 0.005) (Table 3a), while in the R hippocampus (r (22) = 0.39, p = 0.07) and cerebellum (r (23) = 0.35, p = 0.10) the observed correlation was close to statistically significant.

The correlation between the mean dose and SUVmean relative difference was significant in five assessed regions: R hippocampus (r (22) = 0.45, p = 0.03), L occipital lobe (r (23) = 0.52, p = 0.01), L parietal lobe (r (23) = 0.66, p = 0.001), frontal lobe (r (23) = 0.42, p = 0.05), and R occipital lobe (r (23) = 0.55, p = 0.01), and one assessed region, the cerebellum (r (23) = 0.37, p = 0.08), was close to being statistically significant (Table 3a). The correlation between SUVmax absolute difference and maximum dose was significant in five assessed regions: the R hippocampus (r (22) = 0.45, p = 0.04), cerebellum (r (23) = 0.49, p = 0.02), L parietal lobe (r (23) = 0.63, p = 0.001), frontal lobe (r (23) = 0.53, p = 0.01) and L hippocampus (r (23) = 0.45, p = 0.03) (Table 3b).

**Table 3b:** Correlation between SUVmean vs mean dose, SUVmean vs max dose, SUVmax vs mean dose, SUVmax vs max dose absolute and relative difference per assessed region.

Correlations							
		Max_Dose	Mean_Dose	SUV_Mean_Diff_2	RLTV_Mean_Diff	Absoulte_max_SUV_Diff	RLTV_Max_Diff
Max_Dose	Pearson Correlation	1.00	0.76	0.62	0.70	0.53	0.64
	Sig. (2-tailed)		0.00	0.002	0.00	0.01	0.001
	N	23.00	23.00	23.00	23.00	23.00	23.00
Mean_Dose	Pearson Correlation	0.76	1.00	0.34	0.42	0.32	0.41
	Sig. (2-tailed)	0.00		0.12	0.05	0.14	0.05
	N	23.00	23.00	23.00	23.00	23.00	23.00
SUV_Mean_Diff_2	Pearson Correlation	0.62	0.34	1.00	0.97	0.92	0.91
	Sig. (2-tailed)	0.002	0.12		0.00	0.00	0.00
	N	23.00	23.00	23.00	23.00	23.00	23.00
RLTV_Mean_Diff	Pearson Correlation	0.70	0.42	0.97	1.00	0.89	0.94
	Sig. (2-tailed)	0.00	0.05	0.00		0.00	0.00
	N	23.00	23.00	23.00	23.00	23.00	23.00
Absoulte_max_SUV_Diff	Pearson Correlation	0.53	0.32	0.92	0.89	1.00	0.96
	Sig. (2-tailed)	0.01	0.14	0.00	0.00		0.00
	N	23.00	23.00	23.00	23.00	23.00	23.00
RLTV_Max_Diff	Pearson Correlation	0.64	0.41	0.91	0.94	0.96	1.00
	Sig. (2-tailed)	0.001	0.05	0.00	0.00	0.00	
	N	23.00	23.00	23.00	23.00	23.00	23.00
Region = Frontal Lobe							

Correlations							

		Max_Dose	Mean_Dose	SUV_Mean_Diff_2	RLTV_Mean_Diff	Absoulte_max_SUV_Diff	RLTV_Max_Diff

Max_Dose	Pearson Correlation	1.00	0.71	0.37	0.38	0.31	0.22
	Sig. (2-tailed)		0.00	0.08	0.07	0.16	0.32
	N	23.00	23.00	23.00	23.00	23.00	23.00
Mean_Dose	Pearson Correlation	0.71	1.00	0.29	0.30	0.21	0.16
	Sig. (2-tailed)	0.00		0.19	0.16	0.33	0.47
	N	23.00	23.00	23.00	23.00	23.00	23.00
SUV_Mean_Diff_2	Pearson Correlation	0.37	0.29	1.00	0.97	0.83	0.55
	Sig. (2-tailed)	0.08	0.19		0.00	0.00	0.01
	N	23.00	23.00	23.00	23.00	23.00	23.00
RLTV_Mean_Diff	Pearson Correlation	0.38	0.30	0.97	1.00	0.87	0.65
	Sig. (2-tailed)	0.07	0.16	0.00		0.00	0.001
	N	23.00	23.00	23.00	23.00	23.00	23.00
Absoulte_max_SUV_Diff	Pearson Correlation	0.31	0.21	0.83	0.87	1.00	0.89
	Sig. (2-tailed)	0.16	0.33	0.00	0.00		0.00
	N	23.00	23.00	23.00	23.00	23.00	23.00
RLTV_Max_Diff	Pearson Correlation	0.22	0.16	0.55	0.65	0.89	1.00
	Sig. (2-tailed)	0.32	0.47	0.01	0.001	0.00	
	N	23.00	23.00	23.00	23.00	23.00	23.00
Region = Temporal Lobe							

Correlations							

		Max_Dose	Mean_Dose	SUV_Mean_Diff_2	RLTV_Mean_Diff	Absoulte_max_SUV_Diff	RLTV_Max_Diff

Max_Dose	Pearson Correlation	1.00	0.83	0.46	0.43	0.49	0.50
	Sig. (2-tailed)		0.00	0.03	0.04	0.02	0.01
	N	23.00	23.00	23.00	23.00	23.00	23.00
Mean_Dose	Pearson Correlation	0.83	1.00	0.36	0.37	0.39	0.44
	Sig. (2-tailed)	0.00		0.10	0.08	0.07	0.04
	N	23.00	23.00	23.00	23.00	23.00	23.00
SUV_Mean_Diff_2	Pearson Correlation	0.46	0.36	1.00	0.96	0.93	0.93
	Sig. (2-tailed)	0.03	0.10		0.00	0.00	0.00
	N	23.00	23.00	23.00	23.00	23.00	23.00
RLTV_Mean_Diff	Pearson Correlation	0.43	0.37	0.96	1.00	0.88	0.94
	Sig. (2-tailed)	0.04	0.08	0.00		0.00	0.00
	N	23.00	23.00	23.00	23.00	23.00	23.00
Absoulte_max_SUV_Diff	Pearson Correlation	0.49	0.39	0.93	0.88	1.00	0.96
	Sig. (2-tailed)	0.02	0.07	0.00	0.00		0.00
	N	23.00	23.00	23.00	23.00	23.00	23.00
RLTV_Max_Diff	Pearson Correlation	0.50	0.44	0.93	0.94	0.96	1.00
	Sig. (2-tailed)	0.01	0.04	0.00	0.00	0.00	
	N	23.00	23.00	23.00	23.00	23.00	23.00
Region = Cerebellum							

Correlations							

		Max_Dose	Mean_Dose	SUV_Mean_Diff_2	RLTV_Mean_Diff	Absoulte_max_SUV_Diff	RLTV_Max_Diff

Max_Dose	Pearson Correlation	1.00	0.97	0.25	0.25	0.11	0.11
	Sig. (2-tailed)		0.00	0.25	0.25	0.62	0.61
	N	23.00	23.00	23.00	23.00	23.00	23.00
Mean_Dose	Pearson Correlation	0.97	1.00	0.16	0.15	−0.02	−0.01
	Sig. (2-tailed)	0.00		0.47	0.50	0.94	0.96
	N	23.00	23.00	23.00	23.00	23.00	23.00
SUV_Mean_Diff_2	Pearson Correlation	0.25	0.16	1.00	0.97	0.92	0.89
	Sig. (2-tailed)	0.25	0.47		0.00	0.00	0.00
	N	23.00	23.00	23.00	23.00	23.00	23.00
RLTV_Mean_Diff	Pearson Correlation	0.25	0.15	0.97	1.00	0.93	0.95
	Sig. (2-tailed)	0.25	0.50	0.00		0.00	0.00
	N	23.00	23.00	23.00	23.00	23.00	23.00
Absoulte_max_SUV_Diff	Pearson Correlation	0.11	−0.02	0.92	0.93	1.00	0.97
	Sig. (2-tailed)	0.62	0.94	0.00	0.00		0.00
	N	23.00	23.00	23.00	23.00	23.00	23.00
RLTV_Max_Diff	Pearson Correlation	0.11	−0.01	0.89	0.95	0.97	1.00
	Sig. (2-tailed)	0.61	0.96	0.00	0.00	0.00	
	N	23.00	23.00	23.00	23.00	23.00	23.00
Region = R Parietal Lobe							

Correlations							

		Max_Dose	Mean_Dose	SUV_Mean_Diff_2	RLTV_Mean_Diff	Absoulte_max_SUV_Diff	RLTV_Max_Diff

Max_Dose	Pearson Correlation	1.00	0.74	0.51	0.58	0.63	0.62
	Sig. (2-tailed)		0.00	0.01	0.004	0.001	0.002
	N	23.00	23.00	23.00	23.00	23.00	23.00
Mean_Dose	Pearson Correlation	0.74	1.00	0.54	0.66	0.62	0.69
	Sig. (2-tailed)	0.00		0.01	0.001	0.002	0.00
	N	23.00	23.00	23.00	23.00	23.00	23.00
SUV_Mean_Diff_2	Pearson Correlation	0.51	0.54	1.00	0.96	0.90	0.87
	Sig. (2-tailed)	0.01	0.01		0.00	0.00	0.00
	N	23.00	23.00	23.00	23.00	23.00	23.00
RLTV_Mean_Diff	Pearson Correlation	0.58	0.66	0.96	1.00	0.94	0.95
	Sig. (2-tailed)	0.004	0.001	0.00		0.00	0.00
	N	23.00	23.00	23.00	23.00	23.00	23.00
Absoulte_max_SUV_Diff	Pearson Correlation	0.63	0.62	0.90	0.94	1.00	0.97
	Sig. (2-tailed)	0.001	0.002	0.00	0.00		0.00
	N	23.00	23.00	23.00	23.00	23.00	23.00
RLTV_Max_Diff	Pearson Correlation	0.62	0.69	0.87	0.95	0.97	1.00
	Sig. (2-tailed)	0.002	0.00	0.00	0.00	0.00	
	N	23.00	23.00	23.00	23.00	23.00	23.00
Region = L Parietal Lobe							

Correlations							

		Max_Dose	Mean_Dose	SUV_Mean_Diff_2	RLTV_Mean_Diff	Absoulte_max_SUV_Diff	RLTV_Max_Diff

Max_Dose	Pearson Correlation	1.00	0.83	0.50	0.58	0.45	0.54
	Sig. (2-tailed)		0.00	0.02	0.01	0.04	0.01
	N	22.00	22.00	22.00	22.00	22.00	22.00
Mean_Dose	Pearson Correlation	0.83	1.00	0.39	0.45	0.41	0.48
	Sig. (2-tailed)	0.00		0.07	0.03	0.06	0.03
	N	22.00	22.00	22.00	22.00	22.00	22.00
SUV_Mean_Diff_2	Pearson Correlation	0.50	0.39	1.00	0.97	0.60	0.66
	Sig. (2-tailed)	0.02	0.07		0.00	0.003	0.001
	N	22.00	22.00	22.00	22.00	22.00	22.00
RLTV_Mean_Diff	Pearson Correlation	0.58	0.45	0.97	1.00	0.71	0.79
	Sig. (2-tailed)	0.01	0.03	0.00		0.00	0.00
	N	22.00	22.00	22.00	22.00	22.00	22.00
Absoulte_max_SUV_Diff	Pearson Correlation	0.45	0.41	0.60	0.71	1.00	0.97
	Sig. (2-tailed)	0.04	0.06	0.003	0.00		0.00
	N	22.00	22.00	22.00	22.00	23.00	22.00
RLTV_Max_Diff	Pearson Correlation	0.54	0.48	0.66	0.79	0.97	1.00
	Sig. (2-tailed)	0.01	0.03	0.001	0.00	0.00	
	N	22.00	22.00	22.00	22.00	22.00	22.00
Region = R Hippocampus							

Correlations							

		Max_Dose	Mean_Dose	SUV_Mean_Diff_2	RLTV_Mean_Diff	Absoulte_max_SUV_Diff	RLTV_Max_Diff

Max_Dose	Pearson Correlation	1.00	0.99	0.34	0.38	0.45	0.53
	Sig. (2-tailed)		0.00	0.12	0.07	0.03	0.01
	N	23.00	23.00	23.00	23.00	23.00	23.00
Mean_Dose	Pearson Correlation	0.99	1.00	0.31	0.35	0.41	0.48
	Sig. (2-tailed)	0.00		0.15	0.10	0.05	0.02
	N	23.00	23.00	23.00	23.00	23.00	23.00
SUV_Mean_Diff_2	Pearson Correlation	0.34	0.31	1.00	0.98	0.93	0.92
	Sig. (2-tailed)	0.12	0.15		0.00	0.00	0.00
	N	23.00	23.00	23.00	23.00	23.00	23.00
RLTV_Mean_Diff	Pearson Correlation	0.38	0.35	0.98	1.00	0.89	0.93
	Sig. (2-tailed)	0.07	0.10	0.00		0.00	0.00
	N	23.00	23.00	23.00	23.00	23.00	23.00
Absoulte_max_SUV_Diff	Pearson Correlation	0.45	0.41	0.93	0.89	1.00	0.97
	Sig. (2-tailed)	0.03	0.05	0.00	0.00		0.00
	N	23.00	23.00	23.00	23.00	23.00	23.00
RLTV_Max_Diff	Pearson Correlation	0.53	0.48	0.92	0.93	0.97	1.00
	Sig. (2-tailed)	0.01	0.02	0.00	0.00	0.00	
	N	23.00	23.00	23.00	23.00	23.00	23.00
Region = L Hippocampus							

Correlations							

		Max_Dose	Mean_Dose	SUV_Mean_Diff_2	RLTV_Mean_Diff	Absoulte_max_SUV_Diff	RLTV_Max_Diff

Max_Dose	Pearson Correlation	1	0.631	0.327	0.315	0.346	0.338
	Sig. (2-tailed)		0.001	0.128	0.143	0.106	0.114
	N	23	23	23	23	23	23
Mean_Dose	Pearson Correlation	0.631	1	0.56	0.546	0.637	0.59
	Sig. (2-tailed)	0.001		0.005	0.007	0.001	0.003
	N	23	23	23	23	23	23
SUV_Mean_Diff_2	Pearson Correlation	0.327	0.56	1	0.963	0.95	0.934
	Sig. (2-tailed)	0.128	0.01		0	0.00	0.00
	N	23	23	23	23	23	23
RLTV_Mean_Diff	Pearson Correlation	0.315	0.546	0.963	1	0.928	0.983
	Sig. (2-tailed)	0.143	0.01	0		0	0
	N	23	23	23	23	23	23
Absoulte_max_SUV_Diff	Pearson Correlation	0.346	0.637	0.95	0.928	1	0.951
	Sig. (2-tailed)	0.106	0.001	0	0		0
	N	23	23	23	23	23	23
RLTV_Max_Diff	Pearson Correlation	0.338	0.59	0.934	0.983	0.951	1
	Sig. (2-tailed)	0.114	0.003	0	0	0	
	N	23	23	23	23	23	23
Region = R Occipital Lobe							

Correlations							

		Max_Dose	Mean_Dose	SUV_Mean_Diff_2	RLTV_Mean_Diff	Absoulte_max_SUV_Diff	RLTV_Max_Diff

Max_Dose	Pearson Correlation	1	0.854	0.357	0.375	0.348	0.371
	Sig. (2-tailed)		0	0.094	0.077	0.103	0.082
	N	23	23	23	23	23	23
Mean_Dose	Pearson Correlation	0.854	1	0.529	0.517	0.519	0.5
	Sig. (2-tailed)	0		0.01	0.01	0.01	0.02
	N	23	23	23	23	23	23
SUV_Mean_Diff_2	Pearson Correlation	0.357	0.529	1	0.967	0.967	0.937
	Sig. (2-tailed)	0.094	0.01		0	0	0
	N	23	23	23	23	23	23
RLTV_Mean_Diff	Pearson Correlation	0.375	0.517	0.967	1	0.94	0.985
	Sig. (2-tailed)	0.077	0.01	0		0	0
	N	23	23	23	23	23	23
Absoulte_max_SUV_Diff	Pearson Correlation	0.348	0.519	0.967	0.94	1	0.952
	Sig. (2-tailed)	0.103	0.01	0	0		0
	N	23	23	23	23	23	23
RLTV_Max_Diff	Pearson Correlation	0.371	0.5	0.937	0.985	0.952	1
	Sig. (2-tailed)	0.082	0.02	0	0	0	
	N	23	23	23	23	23	23
Region = L Occipital Lobe							

The correlation between the SUVmax relative difference and the maximum dose was significant in the five assessed regions, including the R hippocampus (r (22) = 0.541, p = 0.01), cerebellum (r (23) = 0.50, p = 0.01), L parietal lobe (r (23) = 0.62, p = 0.002), frontal lobe (r (23) = 0.63, p = 0.001), and L hippocampus (r (23) = 0.52, p = 0.01) (Table 3b).

**Fig. 3:**
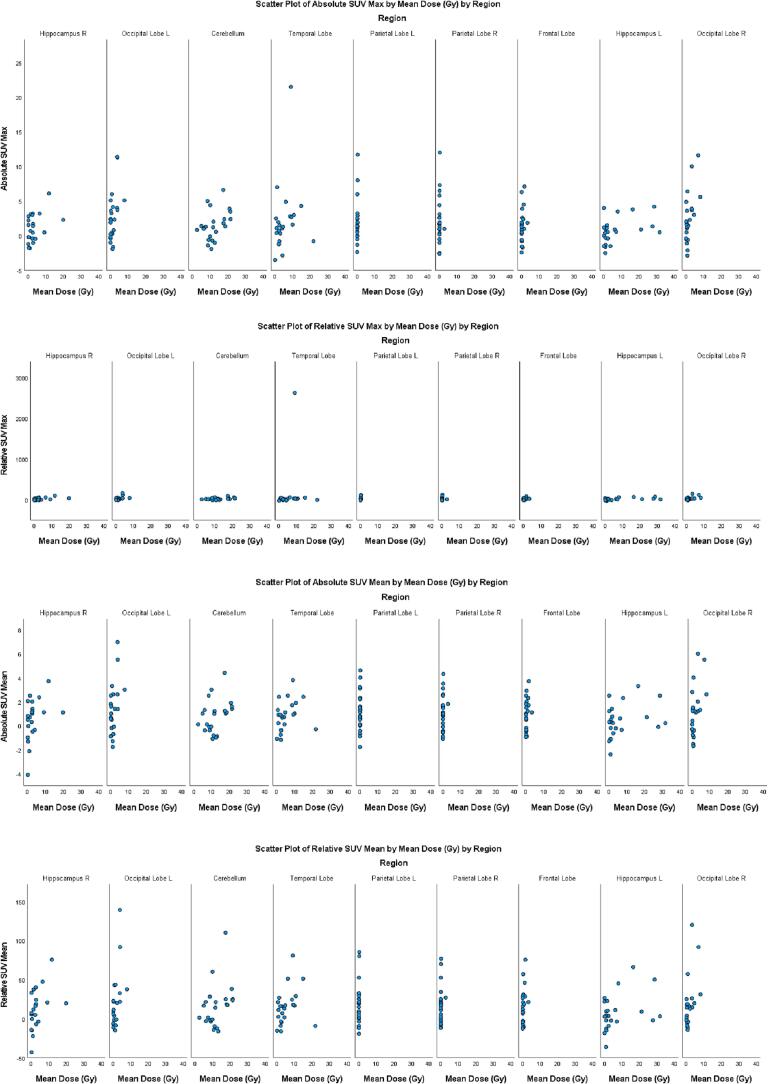
Variation of SUVmean and SUVmax differences vs mean dose for those areas where the Pearson coefficient is >0.5 (<−0.5).

The results, the dose distributions (mean dose and maximum dose) in the selected regions (average, median, interquartile, 10th − 90th percentiles) are also provided (Table 4). Furthermore, the variations in SUVmean and SUVmax absolute and relative differences vs. the mean dose of the assessed brain regions are shown in Fig. 3.

**Table 4:** A Table with dose distribution (mean dose and maximum dose) in the selected regions (average, median, interquartile, 10th-90th percentiles).

Dose_Max	Frontal Lobe	Temporal Lobe	Cerebellum	R Parietal Lobe	L Parietal Lobe	R Hippocampus	L Hippocampus	R Occipital Lobe	L Occipital Lobe
Average	17.53	57.76	43.88	2.48	1.00	9.67	18.30	8.71	8.92
Median	2.94	59.00	41.82	0.21	0.17	9.91	8.45	7.00	10.77
75th. Perc	22.05	67.72	51.56	0.96	0.81	13.38	32.12	12.73	13.73
25th. Perc	0.99	53.87	32.43	0.10	0.11	1.18	4.70	5.31	1.53
90th. Perc	71.24	71.58	67.12	7.52	4.02	24.81	63.26	16.25	16.20
10th. Perc	0.59	38.53	28.15	0.06	0.09	0.23	0.54	1.16	0.28

Dose_Mean	Frontal Lobe	Temporal Lobe	Cerebellum	R Parietal Lobe	L Parietal Lobe	R Hippocampus	L Hippocampus	R Occipital Lobe	L Occipital Lobe

Average	0.47	5.40	12.13	0.17	0.07	3.34	7.13	1.70	1.51
Median	0.09	3.00	11.13	0.03	0.04	2.28	1.82	0.71	0.75
75th. Perc	0.58	9.06	17.51	0.10	0.09	3.08	7.86	3.21	2.20
25th. Perc	0.05	1.99	8.40	0.01	0.02	0.22	0.85	0.26	0.07
90th. Perc	1.52	13.25	21.21	0.19	0.22	10.97	28.25	5.95	3.91
10th. Perc	0.01	0.69	5.40	0.001	0.004	0.07	0.06	0.04	0.02

## Discussion

The objective of our study was to investigate, for the first time, whether regional glucose metabolism, measured using [^18^F]FDG-PET, is altered in non-tumor tissues in the brains of head and neck cancer patients with head and neck cancer after IMPT treatment. Furthermore, we investigated if there is a dose–response relationship in line with the existing literature available for photon radiotherapy. Our main finding was that at 3 months of follow-up after IMPT, the glucose metabolism expressed by SUVmean and SUVmax was higher than before IMPT.

Our measurement time point of 3 months falls approximately between the timepoints of 3 weeks and 6 months observed in the study by Hahn et al. [16], which also was conducted with photon radiotherapy; therefore, it cannot be directly compared with our results. In agreement with Hahn et al., we observed a negative correlation between SUVmean and dose when considering all regions, although at lower dose levels (e.g., see Fig. 3) than the >40 Gy cut-off used in the study by Hahn et al. Some regions of the brain, such as the R hippocampus, cerebellum, and L parietal lobe, showed a statistically significant negative correlation between absolute and relative changes in SUVmean SUVmax in regions that received maximum and mean doses. The findings of the correlation between radiation dose and glucose uptake in our study also align with the preclinical results of Parente et al. [14], which were performed using photon-based radiotherapy. Parente et al. showed that glucose uptake was higher in the brains of rats that received photon cranial radiation at 10 Gy than in those that received 25 Gy. The observed increase in SUV after 10 Gy was attributed to the fact that there may be transient glial cell activation. Cell death or irreversible damage has been emphasized to be more prevalent in the group receiving 25 Gy [14]. Consistent with this, we also observe that at 3 months of radiation dose from proton therapy received by non-tumor brain tissue, an inflammatory component is likely to be reflected in the increased uptake of [^18^F]FDG. Future studies at later timepoints should aim to evaluate at which point (e.g., 6 months of follow-up) this effect diminishes.

In our study, we observed variability between patients and regions, which could draw some parallels regarding similar variations between and even within patients (laterality of brain structures) regarding individual brain structure atrophy in response to radiation dose due to differences in radiation sensitivity. Therefore, future studies on this topic should incorporate high-resolution anatomical data (brain MRI and brain structure volumetric measurements) and PET imaging information of the same regions and structures, to observe the degree of metabolic and anatomical changes in non-tumor brain tissue structures in response to proton radiation dose. According to volumetric studies, even within the same patient, the L and R hippocampi, or the L and R hemisphere, can respond differently to similar amounts of radiation [17], [18], [19].

As we applied FDG-PET at baseline and at 3 months after IMPT, our study provides accurate information about changes in glucose metabolism before and after IMPT in some brain regions related to cognition, such as the temporal lobe and hippocampus**.** However, we found a significant correlation between SUV and radiation dose in all regions of the brain except the R and L hippocampi (p = 0.11 and 0.15, respectively). The correlation in the hippocampal regions probably did not reach statistical significance due to the limited sample size.

Zhang et al. [20] found that low-dose proton therapy was an independent predictor of late damage to the temporal lobe in patients with NPC. Additionally, not only can a low dose have a negative effect on the temporal lobe, but many studies have reported temporal lobe injury in patients receiving a high radiation dose bath for head and neck cancer [8], [21]. As exemplified by Fig. 3, some structures (e.g., Cerebellum and Hippocampus) receive significant dose (up to 40 Gy in some patients) in the context of proton therapy for head and neck cancer, once again stressing the importance to develop better imaging tools to study and promptly identify the effects of this type of radiation on non-tumor tissue.

Furthermore, several studies reported that some brain regions have higher sensitivity to radiation than others; these regions are hippocampus, temporal lobe, and prefrontal cortex [4], [22], [23]. Moreover, scatter radiation has a negative effect on the brain and has been observed in the prefrontal cortex, hippocampus, temporal lobe, and cerebellum [4], [22]. One of the negative effects that might occur in the brain due to scattered radiation are disturbances in the cerebral blood flow. Microvascular damage in hippocampus, cerebellum, and temporal lobe due to radiation effects have been shown to correlate with cognitive deficit [8], [9].

Also, sex of the patient has been suggested to play a role in the severity of responses to radiation. Earlier preclinical studies concluded that the prefrontal cortex in females was more sensitive to low-dose bath than in males [22]. However, this was not the case in our findings; [^18^F]FDG uptake in females was not different from that in males. Furthermore, age also has an effect on cerebral glucose metabolic rate, as some clinical studies reported that [^18^F]FDG uptake in the brains of children was higher than that in adults [24], [25]. Phillips et al. [24] found that pediatric cancer patients receiving cranial radiation therapy had a lower intelligence quotient (IQ) than children treated with chemotherapy alone. Furthermore, in this aforementioned study, younger children treated with radiotherapy had significantly lower IQ test scores than patients aged >18 years. In our study, we did not evaluate the age dependence of [^18^F]FDG uptake due to limited sample size.

Additional clinical studies are needed to investigate the effect of low-dose radiation on the brain using more specific PET tracers, such as 1-(2-chlorophenyl)-N-[^11^C]methyl-(1-methylpropyl)-3-isoquinoline carboxamide [^11^C]PK11195, to detect neuroinflammation. Furthermore, including cognitive tests is important in future studies, which should focus on evaluating specific cognitive domains and correlating cognitive outcomes with SUV changes and the IMPT dose. These findings provide a better understanding of the regional effects of low-dose IMPT on cognition. In addition, implementing artificial intelligence-based tools could improve the sensitivity in detecting neuroinflammation and identifying the sensitive brain structures that could be spared [26]. Moreover, advanced radiotherapy techniques, such as IMPT can be used to actively spare healthy brain tissue as much as possible and further optimize dose to the target volume [27], which in turn can limit the impact of radiation therapy on cognitive function.

## Conclusion

This study demonstrates that [^18^F]FDG-PET imaging can be used to visualize altered glucose metabolism resulting from radiation delivered to the brain, even three months after the completion of IMPT treatment. Changes in the SUV appear to be dependent on the radiation dose in some individual brain regions. Our findings likely reflect a sustained increase in tissue metabolism in response to relatively lower doses of radiation. Future studies including a larger number of patients are needed to further elucidate the sensitivity of different brain structures to proton therapy and evaluate the duration of changes in SUV and their relation to the clinical side effects of IMPT, such as cognitive dysfunction.

## Declaration of Competing Interest

The authors declare that they have no known competing financial interests or personal relationships that could have appeared to influence the work reported in this paper.
