# Supplementary material for: Effects of proton therapy on regional [18F]FDG uptake in non-tumor brain regions of patients treated for head and neck cancer
Source: Clin Transl Radiat Oncol. 2023 Jun 19;42:100652. doi: 10.1016/j.ctro.2023.100652 (PMC10320497; doi:10.1016/j.ctro.2023.100652)
Supplement: Supplementary data 1 [file mmc1.docx]

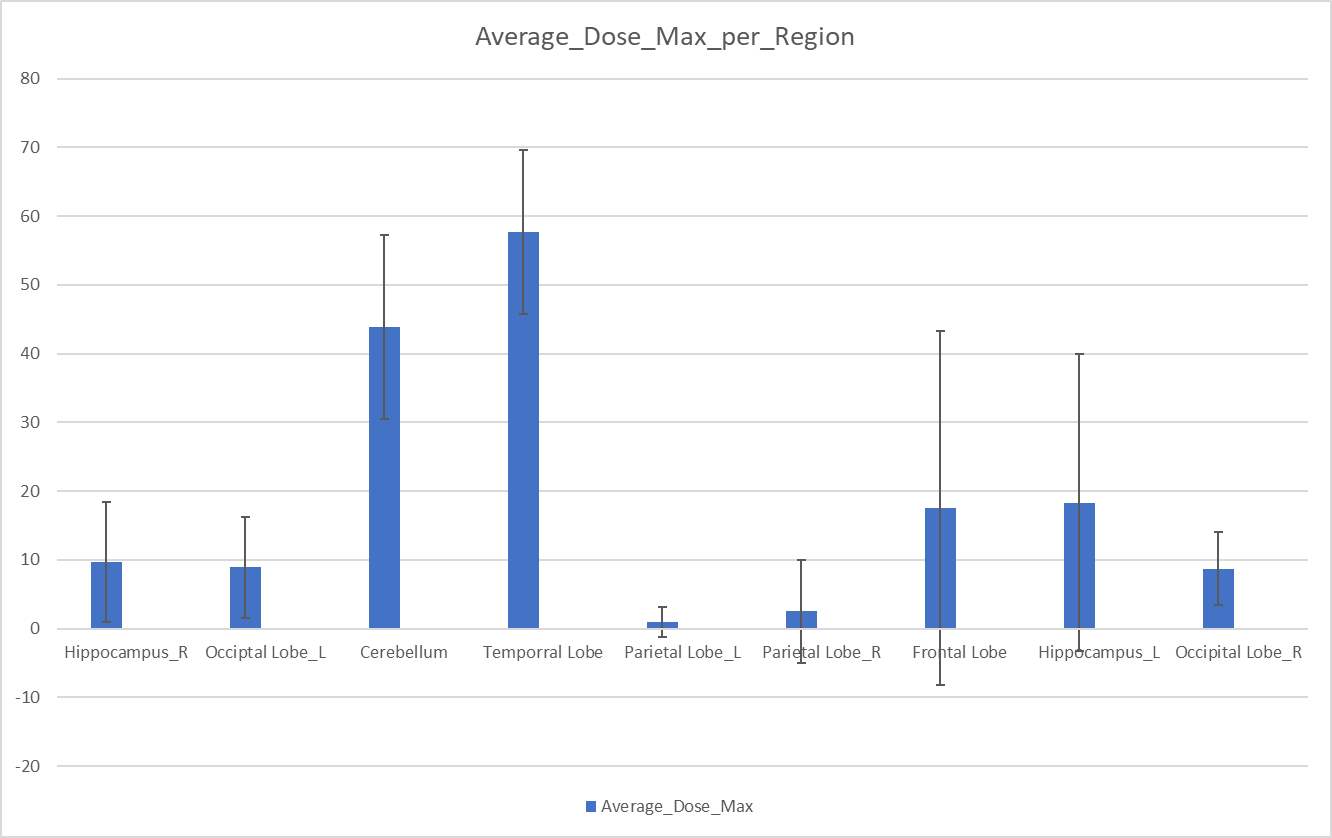


**Average dose max across the assessed regions with the standard deviation**


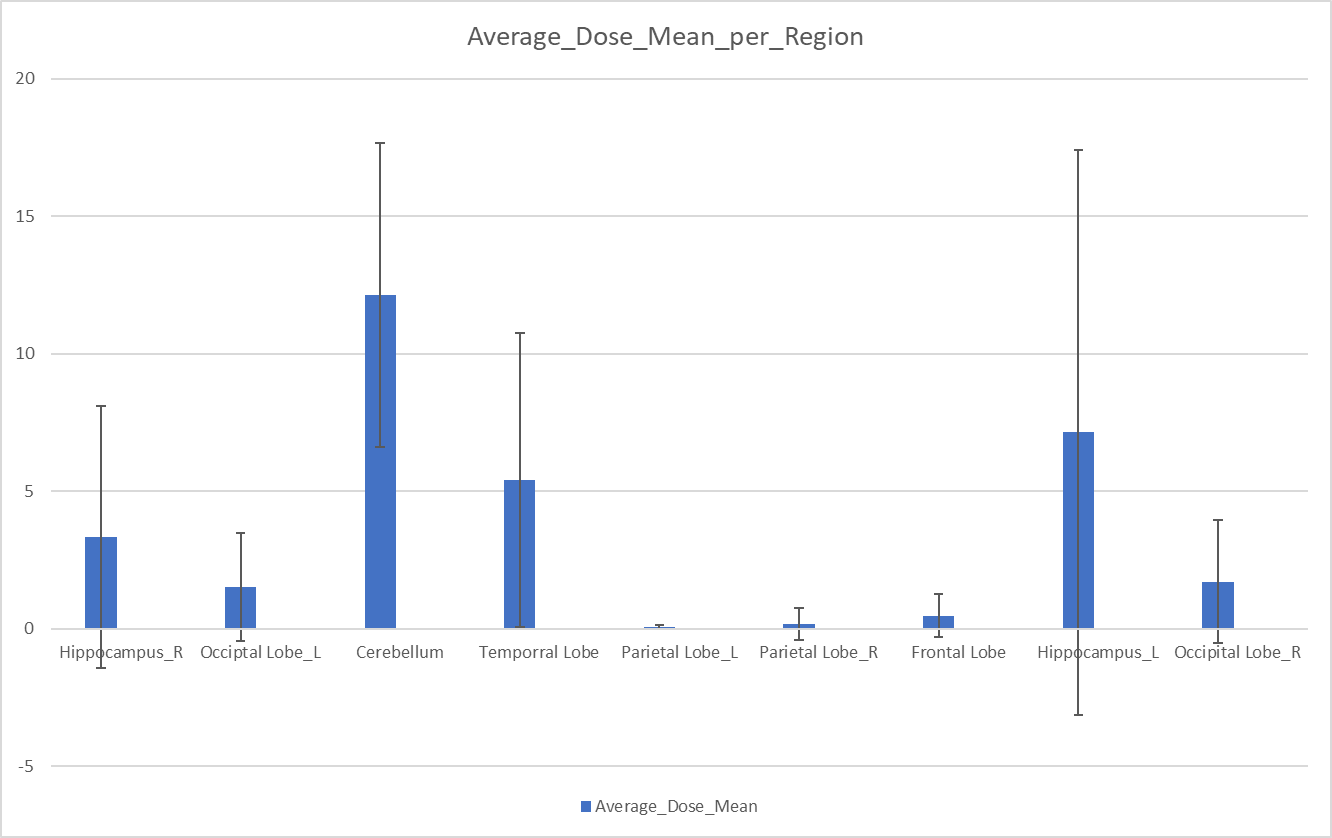


**Average dose mean across the assessed regions with the standard deviation**
